# Surgical Outcomes in Early-Stage Cervical Cancer Following Radical Hysterectomy in a Resource-Limited Setting: The Experience of the National Cancer Institute (Apeksha Hospital, Maharagama), Sri Lanka

**DOI:** 10.7759/cureus.47744

**Published:** 2023-10-26

**Authors:** Yapa Wijeratne, Chintana Hapuachchige

**Affiliations:** 1 Gynecological Oncology, Apeksha Hospital, Maharagama, LKA

**Keywords:** surgery, laparotomy, sri lanka, surgical outcome, cancer survival, early stage, radical hysterectomy, cervical cancer

## Abstract

Introduction

Cervical carcinoma is the commonest gynecological malignancy in Sri Lanka, and this incidence is rising. Radical hysterectomy is the preferred treatment for stages IB1, IB2, and selected cases of stages IIA1. Although surgery is feasible unless the parametrium is involved, careful patient selection is crucial in order to prevent the patient from receiving a dual mode of radical treatment (surgery and radiotherapy). Pre-operative imaging with MRI and PET-CT can accurately determine the tumor size and the lymph node metastasis. In a resource-limited setting, management is challenging as access to MRI and PET-CT is limited. This study was carried out to evaluate the surgical outcomes, five-year survival, and disease-free survival following radical hysterectomies at the National Cancer Institute, Sri Lanka, which performs the largest number of radical hysterectomies annually in the country.

Methods

Seventy-four patients who underwent radical hysterectomy, along with pelvic lymphadenectomy, between July 2015 to January 2017 at the National Cancer Institute, Sri Lanka (Apeksha Hospital Maharagama), were reviewed retrospectively, analyzing their clinical data and histopathological findings. Univariate analysis was performed to identify associated factors and mean survival. The Kaplan-Meier method was used for survival analysis.

Results

With a median follow-up of 63.5 months, five-year disease-free survival was 94.5%, and the five-year overall survival was 95.9%. There is a statistically significant association between mean survival and the cell type, presence of LVSI, and residual tumor on the hysterectomy specimen. Only four patients developed recurrences and four patients died during the follow-up. Eighteen (24.3%) patients needed blood transfusion postoperatively and no cases of bladder or bowel dysfunction in the one year post-operatively. Thirty patients (40.5%) required postoperative adjuvant treatment.

Conclusions

This analysis shows excellent surgical and oncological outcomes following radical hysterectomy as a primary treatment and comparable five-year disease-free survival with available global figures, despite the multiple scarcity of resources. Further studies are needed to assess the national-level status, and limited access to imaging during surgical planning poses a risk for patients receiving dual modes of radical treatment.

## Introduction

In 2020, there were 604,000 new cases and 342,000 deaths that occurred worldwide due to carcinoma cervix, which was the leading cause of gynecological cancer deaths in Southeast Asia [1]. Cervical cancer is the commonest gynecological malignancy in Sri Lanka, and this incidence is rising despite having a known premalignant stage and an established national screening program [2,3]. There are around 1,407 new cases reported annually, while nearly 780 deaths occur due to cervical cancer in Sri Lanka [3]. For nearly 8.8 million female population at risk of cervical cancer [3], 26 tertiary care hospitals island-wide provide initial curative care; however, there are only three specialized gynecological oncology units [4].

Cervical cancer staging is crucial for deciding the primary mode of treatment, hence the outcome. Recent data show the five-year relative survival rate drops from 91.2% to 59.8% among cervical cancer patients who had localized disease to the regionally spread disease [5]. In 2018, the Gynecologic Oncology Committee of the International Federation of Gynecologists and Obstetricians (FIGO) revised the staging of carcinoma cervix to allow the option of clinical, radiological, or pathological findings to assign specific stages where cervical cancer was traditionally staged based on clinical findings [6].

Stage I indicates a tumor strictly confined to the cervix, and stage II indicates the spread beyond the uterus without the involvement of the lower one-third of the vagina or lateral pelvic wall. Stage I-B is sub-staged according to tumor size less than 2 cm, 2-4 cm, and more than 4 cm in stages IB1, IB2, and IB3, respectively [6]. Surgery is the preferred method for the treatment of histologically confirmed early-stage cervical cancer, and modified radical hysterectomy is the treatment for stage I-A1 with lymphovascular space invasion (LVSI) and stage I-A2. Stages IB1, IB2, and IIA1 are usually treated by type C radical hysterectomy with pelvic lymphadenectomy in the absence of fertility wishes [6,7]. Surgery or radiotherapy can be selected as the primary modality of treatment depending on the patient characteristics and local resources in the International Federation of Gynecology and Obstetrics (FIGO) stages IB3 and IIA1 as the outcomes are similar [6]. Stages IA, IB1, IB2, and IIA1 are considered FIGO early stages where primary surgery can be done with a curative intent [6].

Since Ernest Wertheim’s first radical hysterectomy in 1898, he has studied more than 500 patients and published the data in 1911, revealing an 18.6% mortality rate and 42.4% five-year cure rate [8]. However, over the last century, the mortality rate and morbidity have significantly reduced owing to more anatomical knowledge, surgical advances including nerve sparring techniques, and laparoscopic/robotic approaches. Mostly, the data are derived from developed countries, and data are lacking in resource-limited settings [9]. No large numbered data are available in the South East Asia region.

The National Cancer Institute (NCI) (Apeksha Hospital, Maharagama) performs the largest number of radical hysterectomies annually in Sri Lanka. However, there is limited availability for cross-sectional imaging, positron emission tomography-computed tomography (PET-CT), frozen section facilities, and limited space for staged surgeries could lead to a potential need for adjuvant treatment although the dual mode of radical treatment carries higher morbidity.

The objective of the study was to evaluate the surgical outcomes following radical hysterectomy in early-stage invasive cervical cancer carried out at the NCI and five-year overall survival (OS) and disease-free survival (DFS).

## Materials and methods

Seventy-four patients who underwent radical hysterectomy, along with pelvic lymphadenectomy, between July 2015 to January 2017 at the NCI were reviewed retrospectively. Patient details were retrieved from the surgical theatre directory and patient clinical records where operative notes, imaging details, histological reports, and follow-up data including complications and recurrence were analyzed in each patient. Ethical approval was obtained from the Ethical Review Committee, Castle Street Hospital for Women (Teaching), Colombo, Sri Lanka (approval number: ERC/291/08/2021).

The FIGO 2018 staging system was used for assigning the stage depending on the histological findings of tumor size and vaginal and parametrial extension. Lymph node metastasis detected in the histology has not been recorded as stage III C in the analysis as it was not known pre-operatively. Inclusion criteria included all radical hysterectomies carried out for operable cases of carcinoma cervix. All were open abdominal surgeries, and there were no laparoscopic or robotic radical hysterectomies. There were no cases of radical hysterectomy performed during pregnancy. Recurrent cervical cancers, surgery following radiotherapy, radical hysterectomy performed for other gynecological cancers, and patients with any other co-existing cancers were excluded.

For the diagnosis of carcinoma cervix, patients either had a cone biopsy, loop electrosurgical excision procedure (LEEP), or a punch biopsy in larger growths. These patients underwent open radical hysterectomies in the absence of loco-regional extension clinically or radiological, as well as no lymph node involvement in imaging. In the NCI, the radical hysterectomy performed falls into type C1 radical hysterectomy according to Querleu and Morrow’s classification with nerve-sparring [10]. Routine bilateral pelvic lymph node dissection involves common iliac, external iliac, internal iliac, and obturator nodes. Para-aortic lymphadenectomy was not routinely done and was considered only in grossly enlarged or highly suspicious cases. All histological specimens were analyzed by experienced consultant pathologists. Histological type, grade, depth of stromal invasion, parametrial and vaginal margin involvement, LVSI, and lymph node metastasis were considered. Lymph node ultra-staging has not been routinely performed.

Postoperative radiotherapy or concurrent platinum-based chemoradiation received four to five weeks post-operatively for those who meet the criteria for adjuvant treatment [11]. All patients were followed up three monthly for one year, four monthly for the next year, six monthly for up to five years, and annually for 10 years if they received adjuvant treatment. The follow-up method was a clinical examination, vaginal vault cytology smears, and ultrasound imaging. A vaginal vault smear for high-risk HPV DNA was not available. In suspicion of recurrence clinically or in imaging, biopsies were undertaken with or without colposcopy.

Acute (within 30 days following the surgery) and late (after 30 days following the surgery) surgical complications were recorded. A urine culture was not performed as a part of the routine post-operative follow-up care. However, patients have been advised to check urine if they become symptomatic following the hospital discharge with an indwelling urinary catheter. Recurrence was defined as the histologically proven return of cancer after the initial surgery. The duration from surgery to the histological diagnosis of recurrence or disease-specific death, whichever comes first was defined as disease-free survival (DFS), and the date of the surgery to the date of death from any cause was considered as overall survival (OS).

Absolute counts and percentages were used to summarize patient demographic and clinical data. Continuous variables were assessed with mean, standard deviation (SD), independent t-test, and one-way ANOVA test. Categorical variables were assessed using the chi-square test. The Kaplan-Meier method was used to estimate the distribution of time to event endpoints of overall survival and disease-free survival and differences were assessed using the log-rank test for statistical significance. The p-value of <0.05 was considered statistically significant. Statistical Product and Service Solutions (SPSS) (version 25; IBM Corp., Armonk, NY) was used for all analyses.

## Results

Seventy-four patients were included in the study. The mean age was 52.5 (SD: 10.5) years, and the mean parity was 2.59 (SD: 1.17). There were 14 diabetics (18%), 20 hypertensives (27%), and one (1.4%) ischemic heart disease patient, and there was no statistically significant association with mean survival and the presence of diabetes (p=0.257) or hypertension (p=0.921).

Only four patients had MRIs, and 22 had CT abdomen and pelvis pre-operatively. All patients had an ultrasound scan abdomen and pelvis. However, no patient had a pre-operative PET-CT.

All patients underwent radical hysterectomy and pelvic lymphadenectomy. No patient had para-aortic lymphadenectomy. The mean number of lymph nodes removed was 7.87 (SD: 3.35, range: 2-21). Lymph nodes were positive for tumor metastasis only in seven (9.4%) patients.

A statistically significant association has been shown between the mean survival and the cell type, presence of LVSI, and residual tumor on the hysterectomy specimen (Table 1). However, there was no association between the LVSI and deaths (p=0.55).

**Table 1:** The results of univariate analysis of the association between the mean survival and clinicopathological variables The results of univariate analysis of the association between the mean survival and clinicopathological variables (including age, status of salpingo-oophorectomy performed, histological cell type, LVSI, lymph node status-positivity for tumor cells, vaginal margin status positivity for tumor cells, presence of residual tumor in the uterine specimen and adjuvant treatment) *The test was performed on squamous vs adenocarcinoma vs small cell/neuroendocrine Abbreviations: LVSI: lymph-vascular space invasion

Characteristic	Number (%)	Mean Survival in Months (SD)	P value
Age (y)			0.578
<50	33 (44.6%)	54.24 (22.1)	
> 51	41 (55.4%)	59.17 (21.21)	
Salpingo-oophorectomy			0.221
None	10 (13.5%)	52.9 (18.08)	
Unilateral	9 (12.2%)	46.78 (24.86)	
Bilateral	55 (74.3%)	59.38 (21.41)	
Cell type			0.042*
Squamous cell carcinoma	61 (82.4%)	57.01 (21.36)	
Adenocarcinoma	12 (16.2%)	61.08 (18.51)	
Small cell/neuroendocrine	1 (1.4%)	5	
Stage			0.464
IA2	17 (23.0%)	57.88 (19.29)	
IB1	16 (21.5%)	66.31 (14.03)	
IB2	25 (33.8%)	52.68 (26.36)	
IB3	9 (12.2%)	54.22 (25.12)	
II A	6 (8.1%)	53.83 (15.76)	
II B	1 (1.4%)	43	
Grade			0.055
Well	15 (20.2%)	68.60 (17.18)	
Moderate	49 (66.2%)	53.37 (21.39)	
Poor	10 (13.6%)	57.20 (24.28)	
LVSI			0.042
Negative	63 (84.6%)	59.38 (19.46)	
Positive	11 (15.4%)	43.18 (28.50)	
Lymph node status			0.546
Negative	67 (90.6%)	57.42 (21.33)	
Positive	7 (9.4%)	52.71 (25.46)	
Vaginal margin involvement			0.121
Negative	67 (90.6%)	56.58 (22.42)	
Positive	7 (9.4%)	60.71 (11.48)	
Residual tumor in the uterine specimen			0.049
Negative	51 (68.9%)	61.74 (18.82)	
Positive	23 (31.1%)	46.39 (23.95)	
Adjuvant treatment			0.101
Yes	30 (40.5%)	57.50 (16.64)	
No	44 (59.5%)	56.61 (24.60)	

There was only one (1.3%) patient with a parametrial involvement in the histological specimen. No patient had ovarian metastasis.

Regarding post-surgical complications, 18 (24.3%) patients needed blood transfusion postoperatively secondary to the surgical blood loss. There were two (2.7%) cases of postoperative fever, three (4%) wound infections, and three (4%) vaginal bleeding, but no cases of deep vein thrombosis. There were no cases of intra-operative deaths, return to the theatre, or re-admissions. No patient had urinary tract infections and no cases of bladder or bowel dysfunction in the one year post-operatively. Lymphedema was the commonest (n=15, 20.2%) late complication, and one had lymphocyst formation.

Thirty patients (40.5%) required postoperative adjuvant treatment, and lymph node positivity (n=7) and microscopically positive margins (n=7) are the commonest indications, while the main reason (n=15, 50%) is due to a combination of risk factors. Of them, 12 patients (16.2%) received vaginal brachytherapy, while the rest (n=18, 24.3%) received concurrent chemoradiation with cisplatin. In stages IB3 and IIA, 66%, (n=10/15) of them needed to have adjuvant treatment. Two patients (2.7%) had a uretero-vaginal fistula, and one had radiotherapy-induced urethral stricture, which required surgical repair. There was one case of incisional hernia and radiotherapy-induced proctitis. One patient developed chemotherapy-related acute myeloid leukemia in 26 months.

Only four patients (5.4%) developed recurrences, and all of them were in the vaginal vault. The median duration for recurrence is 8.5 months (range: 5-21) (Table 2). There was no association between cancer recurrence and adjuvant treatment (p=0.69) or lymph node metastasis (p=0.27). Of the four deaths, three patients died due to non-disease-specific causes, including two cases of myocardial infarctions. Only the cancer-related death patient had clinical stage IB3 and postoperatively received adjuvant chemoradiation due to positive lymph nodes. She had vault recurrence 10 months post-operative and was complicated with vesicovaginal fistula and renal failure causing the death.

**Table 2:** Characteristics of the cancer recurrence This table shows the clinic-pathological characteristics (age, cell type, initial stage, grade, LVSI, status of oophorectomy performed, lymph node positivity for tumor cells, status of adjuvant treatment received, diagnosis of recurrence since the primary surgery and whether they were alive or not at the end of the study period ) of all four patients who had cancer recurrence. Abbreviations: SCC: squamous cell carcinoma, LVSI: lymph-vascular space invasion, BSO: bilateral salpingo-oophorectomy

Patient	Age	Cell Type	Stage	Grade	LVSI	Oophorectomy Status	Lymph Nodes	Adjuvant Treatment	Recurrence-Free Interval Since Surgery (in Months)	Outcome
1	54	SCC	IB3	3	No	BSO	Positive	Yes	10	Died due to renal failure
2	76	SCC	IB1	1	No	BSO	Negative	No	5	Alive
3	40	SCC	IB3	2	No	BSO	Negative	No	7	Alive
4	62	SCC	IB1	2	No	BSO	Negative	Yes	21	Alive

**Figure 1:**
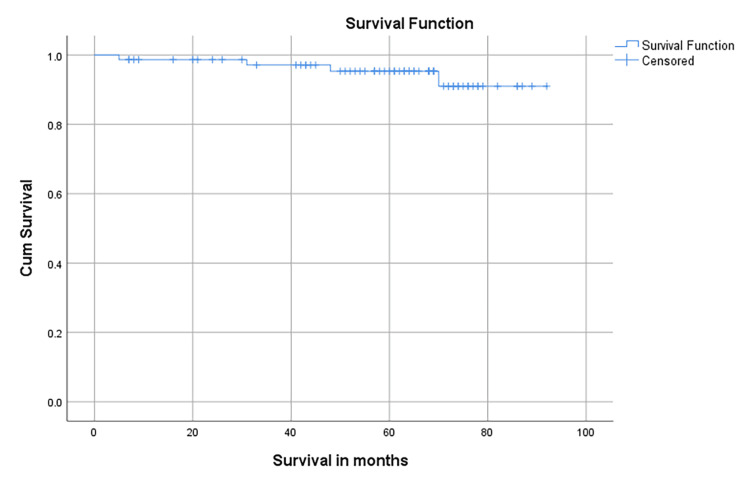
The probability of five-year overall survival following the primary treatment Primary treatment includes radical hysterectomy with or without adjuvant treatment

**Figure 2:**
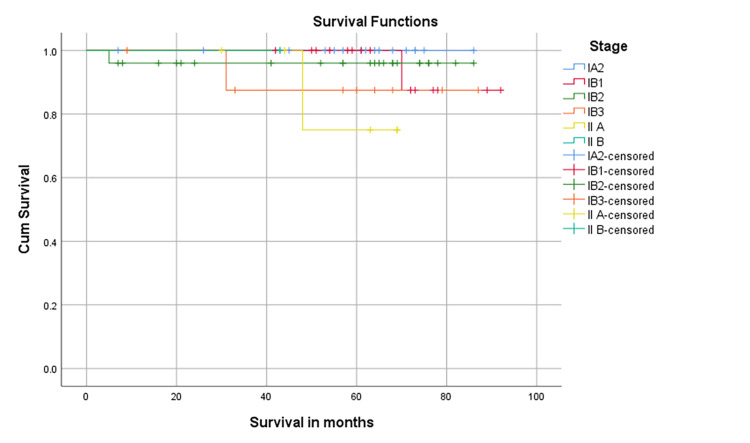
The probability of five-year overall survival following the primary treatment according to the cancer stage Primary treatment includes radical hysterectomy with or without adjuvant treatment

With a median follow-up was 63.5 months (95% CI 58.08- 67.92), the five-year DFS was 94.5%, and the five-year OS was 95.9% (Figure 1). There is no statistical difference between stages IA2, IB1, IB2, IB3, II A, and II B in terms of OS (Log-rank test p=0.509) (Figure 2).

## Discussion

This analysis shows excellent surgical outcomes following radical hysterectomy as a primary treatment and comparable five-year disease-free survival with available global figures [5]. There is a statistically significant association between mean survival and the cell type, presence of LVSI, and residual tumor on the hysterectomy specimen. The NCI follows the FIGO and Sri Lanka College of Obstetricians and Gynaecologist (SLCOG) guidance for the case selection for a radical hysterectomy with pelvic lymphadenectomy [6,12]. In stages IB3 and IIA1, the decision is individualized on patient factors; however, surgery is offered commonly as a primary treatment caused by delays in radiotherapy due to the limited number of radiotherapy units [13].

The positive LVSI has been shown to be an adverse prognostic factor [14]. In this study, 11 (15.4%) patients had positive LVSI and showed a statistically significant association with mean survival. Systematic pelvic lymphadenectomy is an important step in surgical management since the nodal spread is the single most important prognostic factor [6,15]. Although the number of lymph nodes that need to be examined in a lymphadenectomy for cervical cancer is not standardized, the EORTC-GCG study opinion is to remove at least 11 pelvic lymph nodes as a surgical quality indication [16]. However, a recent population-based study has shown that at least eight lymph nodes need to be examined for prognostic stratification [17]. The mean number of lymph nodes removed was 7.87 (range: 2-21), which is below the usual cutoff. The anatomy of the patient, the extent of surgery, the status of local inflammation, specimen processing, and examination have been shown as factors determining the variation in lymph node number [18]. In addition, no further attempts were made to do full lymphadenectomy in cases where enlarged suspicious nodes were observed.

The incidence of lymph node metastasis in stage IA2 with LVSI to stage IIA rises from 4% to 25%, which was comparable to our figures although the sample size is small [19]. Cervical adenocarcinoma (AC) and neuroendocrine tumors have shown poor prognosis compared to squamous cell carcinoma (SCC) [20,21]. However, in this study, all recurrence cases had SCC, and AC has shown a better mean survival.

A cone biopsy or LEEP could be diagnostic for cervical carcinoma, as well as curative in small tumor sizes and residual cancer, could remain in the cervix following these procedures. The presence of residual tumor in the radical hysterectomy specimen following the initial excision procedure is indicative of a higher-stage disease [12]. It has shown that there is no association between open or minimally invasive surgical approaches in no residual disease in early cervical cancer hysterectomy specimens [22,23]. Although all cases are done in an open approach in our study, the presence of residual disease has shown a significantly reduced mean survival.

Ovarian metastasis is observed commonly among AC compared to the SCC (5.3% vs. 0.79%) and has shown a positive correlation with the cancer stage [24,25]. There was no ovarian metastasis in the oophorectomized patients in this study. The parametrial invasion is a contraindication for surgical treatment. The detection of parametrial invasion by clinical examination has a sensitivity ranging from 29% to 66% with specificities from 81% to 99% [26]. Since the MRI facility was limited, the clinical parametrial assessment was carried out by an experienced gynecological oncologist. Only one patient was found to have parametrial involvement in the histopathological specimen.

The presence of a tumor at the resection margin or more than one node with metastatic infiltration or extracapsular nodal spread is considered for postoperative adjuvant chemoradiotherapy or radiotherapy according to the current Sri Lankan guidelines [11]. In cases where the lymph nodes are negative, the positivity of either two of the following factors, namely, LVSI, deep stromal invasion, poorly differentiated (grade 3) tumor, tumor diameter of >4 cm, or invasive tumor less than 5 mm from the resection margin, is also considered the indication for adjuvant treatment [11]. Evidence suggests that around 28% of patients required adjuvant treatment following radical surgery for early cervical cancer and the combination of risk factors for the consideration for adjuvant treatment is as high as 81% [27]. In this study, a higher percentage (n=30, 40.5%) required postoperative adjuvant treatment, but the combination of risk factors as the indication is only 50%, and lymph node positivity and microscopically positive margins are the commonest single indications.

A significant reduction in postoperative blood transfusion need was observed in the 1990s (44%) compared to the 1980s (91%) [28]. In a recent meta-analysis, Geetha et al. [29] have shown that the even lower figures of the median percentage of blood transfusion for abdominal RH is 25%, and this study's figures are 24.3% despite local policy having a lower threshold for post-operative blood transfusions. European data suggest that lymphedema following RH is approximately 25%, and the comparatively low rate (19.2%) observed in this study could be due to the practice of strict wearing of postoperative leg stocking for six months or the variation of lymph nodes removed [30].

Post-operatively, the patients are kept on an indwelling urinary catheter for 10-14 days. However, no cases of culture-proven urinary tract infections were reported. This could possibly be due to the prophylactic use of antibiotics on the discharge for five to seven days as per the unit's local protocol. Post-radical hysterectomy lower urinary tract dysfunction incidence varies from 8% to 80%, and spontaneous recovery is expected in six to 12 months [9]. Nerve sparring radical hysterectomy is carried out in NCI, and no patient had significant bladder or bowel dysfunctions one year post-operatively.

Since all patients were operated and managed on at the same institute, there are several strengths of this study, including the minimal variation of surgical techniques and expert opinion on pathologic slides, including immunohistochemistry. In addition, the follow-up protocol was similar, and the long-term follow-up was maintained. Although this study is based on unit experience, patient representation is from all parts of the country. A few limitations are the relatively small sample size and no available data on the risk factors, body mass index, performance status, and post-operative sexual dysfunction.

In a resource-limited setting, the management of complicated cancer patients is extremely challenging due to difficulty in the diagnosis, staging, and planning of the management. The NCI gets referrals from all over the country, and often the full pathologic description is not mentioned, especially the LVSI, which is an important determinant of surgery. In most cases, re-evaluation of the specimen is also not feasible due to barriers in communication between the distant referring units and extremely limited resources in peripheral units where initial biopsies were done (e.g., no access to immunohistochemistry and lack of expert pathologists for second opinions). Hence, the radicality of surgery is based on other factors, including tumor size and grade. MRI and PET-CT are crucial to staging cervical cancer in terms of better evaluation of the estimation of tumor size, loco-regional involvement, and nodal spread [6]. In expert hands, ultrasonic diagnostic accuracy has shown good results [6], and the nodal involvement has been assessed based on ultrasound, CT, and MRI. A staged surgical procedure (i.e., laparoscopic lymphadenectomy followed by a radical hysterectomy) or frozen section for lymph node status is also an alternative as access to PET-CT is limited, yet again it becomes difficult due to limited operating theatre facilities.

## Conclusions

In conclusion, despite the multiple scarcity of resources, the OS is excellent with good surgical and oncological outcomes. Most patients tolerated the surgery well, and a relatively lower rate of post-surgical and post-radiation-related complications was observed. Further studies are needed to assess the national-level status, and limited access to imaging during surgical planning poses a risk for patients receiving dual modes of radical treatment.
